# CHD7 regulates craniofacial cartilage development via controlling HTR2B expression

**DOI:** 10.1093/jbmr/zjae024

**Published:** 2024-02-04

**Authors:** Maximilian Breuer, Maximilian Rummler, Jaskaran Singh, Sabrina Maher, Charlotte Zaouter, Priyanka Jamadagni, Nicolas Pilon, Bettina M Willie, Shunmoogum A Patten

**Affiliations:** Institut National de la Recherche Scientifique (INRS) – Centre Armand Frappier Santé Biotechnologie, Laval, QC H7V 1B7, Canada; Research Centre, Shriners Hospital for Children-Canada, Department of Biological and Biomedical Engineering, Faculty of Dental Medicine and Oral Health Sciences, McGill University, Montreal H4A 0A9, Canada; Institut National de la Recherche Scientifique (INRS) – Centre Armand Frappier Santé Biotechnologie, Laval, QC H7V 1B7, Canada; Institut National de la Recherche Scientifique (INRS) – Centre Armand Frappier Santé Biotechnologie, Laval, QC H7V 1B7, Canada; Research Centre, Shriners Hospital for Children-Canada, Department of Biological and Biomedical Engineering, Faculty of Dental Medicine and Oral Health Sciences, McGill University, Montreal H4A 0A9, Canada; Département de Neurosciences, Université de Montréal, Montréal, QC H3C 3J7, Canada; Institut National de la Recherche Scientifique (INRS) – Centre Armand Frappier Santé Biotechnologie, Laval, QC H7V 1B7, Canada; Institut National de la Recherche Scientifique (INRS) – Centre Armand Frappier Santé Biotechnologie, Laval, QC H7V 1B7, Canada; Molecular Genetics of Development Laboratory, Départment des Sciences Biologiques, Université du Québec à Montréal (UQAM), Montréal, QC H3C 3P8, Canada; Centre d'Excellence en Recherche sur les Maladies Orphelines - Fondation Courtois (CERMO-FC), Université du Québec à Montréal (UQAM), Montréal, QC H3C 3P8, Canada; Research Centre, Shriners Hospital for Children-Canada, Department of Biological and Biomedical Engineering, Faculty of Dental Medicine and Oral Health Sciences, McGill University, Montreal H4A 0A9, Canada; Institut National de la Recherche Scientifique (INRS) – Centre Armand Frappier Santé Biotechnologie, Laval, QC H7V 1B7, Canada; Département de Neurosciences, Université de Montréal, Montréal, QC H3C 3J7, Canada; Centre d'Excellence en Recherche sur les Maladies Orphelines - Fondation Courtois (CERMO-FC), Université du Québec à Montréal (UQAM), Montréal, QC H3C 3P8, Canada

**Keywords:** diseases and disorders of/related to bone, genetic research, cells of bone

## Abstract

Mutations in the Chromodomain helicase DNA-binding protein 7 – coding gene (*CHD7*) cause CHARGE syndrome (CS). Although craniofacial and skeletal abnormalities are major features of CS patients, the role of CHD7 in bone and cartilage development remain largely unexplored. Here, using a zebrafish (*Danio rerio*) CS model, we show that *chd7^-/-^* larvae display abnormal craniofacial cartilage development and spinal deformities. The craniofacial and spine defects are accompanied by a marked reduction of bone mineralization. At the molecular level, we show that these phenotypes are associated with significant reduction in the expression levels of osteoblast differentiation markers. Additionally, we detected a marked depletion of collagen 2α1 in the cartilage of craniofacial regions and vertebrae, along with significantly reduced number of chondrocytes. Chondrogenesis defects are at least in part due to downregulation of *htr2b*, which we found to be also dysregulated in human cells derived from an individual with *CHD7* mutation-positive CS. Overall, this study thus unveils an essential role for CHD7 in cartilage and bone development, with potential clinical relevance for the craniofacial defects associated with CS.

## Lay Summary

Patients with CHARGE syndrome exhibit skeletal defects. CHARGE syndrome is primarily caused by mutations in the chromatin remodeler-coding gene *CHD7*. To investigate the poorly characterized role of CHD7 in cartilage and bone development, here, we examine the craniofacial and bone anomalies in a zebrafish *chd7^-/-^* mutant model. We find that zebrafish mutant larvae exhibit striking dysmorphism of craniofacial structures and spinal deformities. Notably, we find a significant reduction in osteoblast, chondrocyte, and collagen matrix markers. This work provides important insights to improve our understanding of the role of *chd7* in skeletal development.

## Graphical Abstract

**Graphical Abstract:**
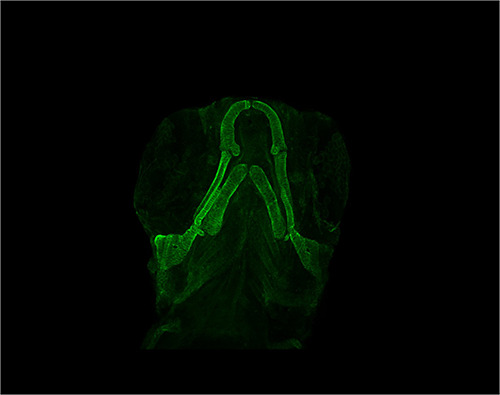

## Introduction

Mutations in the chromodomain helicase DNA binding protein 7- coding gene (*CHD7*) are the major cause of the congenital disorder CHARGE syndrome (CS).^1^ This genetic disorder affects 1 in 10 000 newborns worldwide. CHARGE is an acronym for Coloboma, Heart defects, Atresia of choanae, Retarded growth, Genital and Ear abnormalities.^2-4^ The clinical presentation of CS is highly variable and additional clinical features have been continuously documented to include supplemental craniofacial abnormalities (other than choanal atresia and ear defects) as well as spinal deformations (scoliosis, kyphosis and hemivertebrae).^5-7^ Although these supplemental defects are not considered for clinical diagnosis of CS, they are nonetheless very common.^8^ In fact, idiopathic scoliosis is observed in a majority of CS cases, with studies reporting that over 60% of patients may be affected.^9-11^ Furthermore, the range of craniofacial defects seen in CS is quite broad, frequently including a square face, wide forehead, facial asymmetry, small mouth and chin, cleft lip/palate, in addition to the cardinal features of choanal atresia and ear anomalies.^12^^,^^13^ Abnormalities of the skull base and craniocervical junction^14^ as well as reduced areal bone mineral density (aBMD) have also been reported.^15^ All these reports strongly suggest an important role for CHD7 in the development and functioning of bone and cartilage. However, the mechanisms underlying the skeletal defects in CS remain poorly understood.

Studies in Drosophila, Xenopus, and zebrafish have proven useful in unraveling the roles of CHD7, especially in neural crest cells.^16-18^ Analysis of pathways related to CHD7 in genetic mouse models and patient-derived IPSCs show involvement in neural crest differentiation/proliferation/migration and stem cell quiescence.^19-21^ Furthermore, studies focusing on CS using patient samples and model systems have identified regulatory mechanisms of CHD7 in the immune response and more strikingly in brain development.^22^^,^^23^ Most recently, CHD7 has been connected to abnormal GABAergic development resulting in an autistic-like behavior in a zebrafish model.^24^ While the function of CHD7 has been extensively studied, little attention has been given to its role in skeletal development.^25^ Some studies have directly linked CHD7 to osteogenesis. ^(^^26^^,^^27^^)^ For instance, depletion of CHD7 in human and mouse mesenchymal stem cells (MSCs) inhibit osteogenic transcription factors and impair their differentiation into osteoblasts.^26^^,^^27^ Lui et al.^28^ recently showed that CHD7 regulates osteogenesis in mouse MSCs by suppressing the PPARγ signalling pathway. Various models to investigate this role of CHD7 in vivo have been proposed, including numerous mouse lines. However, mouse models with constitutive CHD7 deficiency have limitations, the main one being the embryonically lethality of homozygous mutants by 10 days post-conception.^29^ Additionally, heterozygous *Chd7* mutant mice present highly variable phenotypes, and the full spectrum and severity of certain CS malformations are not seen.^30^^,^^31^ For instance, *Chd7^Looper/+^* presents ear ossicle malformations but no spinal deformities,^32^ whereas *Chd7^Gt/+^* mice display normal skeletal development.^30^ Hence, multiallelic conditional approaches based on the Cre/LoxP system are needed to consistently uncover skeletal defects, but only a subset at a time. For example, homozygous deletion of a floxed allele of *Chd7* in subsets of ectodermal and endodermal derivatives with the *Foxg1*-*Cre* driver specifically affects nasal cavities and tracheal rings,^30^ whereas targeted deletion in neural crest cells with the *Wnt1*-*Cre* driver specifically leads to cleft palate, among other partially overlapping phenotypes.^30^ In comparison, modelling CS-associated skeletal defects in zebrafish appears simpler, for the main reason that constitutive loss of *chd7* is not embryonic lethal. Notably, zebrafish *chd7-*deficient larvae have been reported to display craniofacial malformations as well as defective vertebrae mineralization.^24^^,^^33^^,^^34^ However, the mechanisms underlying these skeletal phenotypes in *chd7* fish mutants remain elusive.

Zebrafish have become increasingly relevant in the study of bone development and bone-related disorders,^35-37^ including scoliosis, osteoporosis and age-related osteoarthritis.^38^^,^^39^ This vertebrate model organism offers specific advantages in the analysis of skeletal development,^40-43^ including extensive similarities with the human skeleton. We previously reported the development of a zebrafish *chd7* mutant that mimics phenotypes found in individuals with CS.^24^ Here, we use this model to provide a detailed characterization of its skeletal anomalies and to determine the underlying mechanisms of CHD7. Early larvae screens revealed a striking dysmorphism of craniofacial structures and delayed mineralization of vertebral bodies. These defects are associated with problems with both osteogenesis and chondrogenesis, at least in part due to transcriptional downregulation of the 5-hydroxytryptamine (serotonin) receptor 2b gene, *htr2b*.

## Materials and methods

### Zebrafish husbandry

Wildtype (*chd7^+/+^*) and mutant (*chd7^iaf17^*) zebrafish were kept at 28°C in a 12 h/12 h dark/light cycle and maintained in accordance with Westerfield et al.^44^ The *chd7^iaf17^* mutant line (here after referred *as chd7^-/-^*) bearing a single nucleotide insertion causing a frame-shifting mutation was generated using CRISPR/Cas9.^24^ This mutation causes a premature stop codon 8 amino acids after the mutation site and the mutant *chd7* transcript underwent nonsense-mediated decay upon that mutation.^24^ Noteworthy, we did not observe any abnormalities in *chd7* heterozygous mutants including at the level of skeletal phenotypes in our first report on the characterization of the zebrafish *chd7* mutant line.^24^ This is likely due to some teleost-specific genetic compensation mechanisms occurring in heterozygous background.^24^

Genotyping of *chd7^+/+^* (wild-type), *chd7^+/-^* (heterozygous), and *chd7^-/-^* (homozygous) fish was performed by high resolution melting analysis using genomic DNA extracted by boiling larva/clipped caudal fin in 50 mM NaOH for 10 min and then neutralizing it with 100 mM Tris HCl (pH 8).

All zebrafish in this study were housed in groups and fed a steady diet of Skretting®GemmaMicro starting at 5 dpf. Embryos were raised at 28.5°C and staged as previously described by Kimmel et al.^45^ All experiments were performed in line with the guidelines of the Canadian Council for Animal Care and the local ethics committee (#1605-01 and #2005-01).

### Skeletal stainings and analysis of larvae

Alcian blue staining was performed on zebrafish larvae according to Walker and Kimmel.^46^ Alizarin Red was performed on zebrafish larvae (6 dpf, 9 dpf, 12 dpf and 4 weeks) that were fixed with 4% paraformaldehyde (PFA) overnight at 4°C. After fixation, the fish were incubated in 70% ethanol overnight then washed for 1 h in 95% ethanol. After dehydration, samples were fixed in acetone overnight at room temperature and then stained with 0.05% Alizarin Red (Sigma-Aldrich) in 95% ethanol for 3-4 h. After staining, samples were bleached in 1% KOH overnight and stored in 50% glycerol in 0.1% KOH. Larvae were then imaged using a Zeiss stereomicroscope.

### MicroCT Imaging of the spinal and craniofacial skeleton

Adult zebrafish were euthanized using tricaine (MS-222, Sigma-Aldrich). Tissue was fixed in 4% PFA prior to microCT analysis. *Ex vivo* microCT at an isotropic voxel size of 10.6 μm (SkyScan1276, Bruker, Kontich, Belgium) was performed to assess differences in Weberian and vertebral bone mass, mineral density, and microstructure (55 kVp, Al 0.25 mm filter, 200 μA source current, 0.3° steps for full 360°). Additionally, *ex vivo* microCT of craniofacial bone structures at an isotropic voxel size of 5.0 μm was performed (55 kVp, Al 0.25 mm filter, 72 μA source current, 0.16° steps for full 360°) to assess morphological features of the skull. Images were reconstructed using standard reconstruction algorithms provided with the microCT. Spinal and craniofacial deformities of *chd7^+/+^* and mutants were analyzed of 1-year (n = 8 for vertebrae, n = 5 for skull) and 2-year-old (n = 5) zebrafish.

### Analysis and bone density/volume/angles

From the reconstructed microCT images of the whole spine, results from regions of interest were segmented and analyzed, including the Weberian apparatus, as well as the third pre-caudal and sixth caudal vertebrae. Further, the intercalarium, the tripus as well as the parapophysis were segmented from the Weberian apparatus. For the vertebrae, the neural as well as the hemal arch were segmented from the vertebral body. To differentiate between bone and background, a density-based threshold of 0.41 gHA/cm^3^ determined by Otsu’s method^47^ was used. For each segmented bone in the Weberian apparatus outcomes included: bone volume BV (mm^3^), tissue volume TV (mm^3^), bone volume fraction BV/TV (mm^3^/mm^3^), and volumetric bone mineral density vBMD (gHA/cm^3^). For pre-caudal and caudal vertebrae the outcomes included: bone volume BV, tissue volume TV, bone volume fraction BV/TV, total bone mineral density T.vBMD, vertebral body vBMD VB.BMD, vertebral arch vBMD VA.BMD, as well as arch-body angle, body angle, arch opening angle and rising angles. For caudal vertebrae, the angle outcomes were calculated for hemal as well as neural arches. Craniofacial outcomes included: mandibular arch length, complete skull length measured from the tip of the dentary to the furthest point of the supraoccipital, width of the skull, measured between the opercular tips as well as mandibular arch angle, and craniofacial angle measured between dentary and frontal bone (See illustration in Supplementary Figure S1C).

### Quantitative reverse transcription polymerase chain reaction (qRT-PCR)

Total RNA was isolated from 9 dpf and 12 dpf old zebrafish larvae (N = 3, n = 30) using TriReagent. 1 μg of RNA was used for cDNA synthesis using cDNA vilo kit (ThermoFisher). qRT-PCR was performed with SYBR Green mix (BIORAD) with a Lightcycler96® (Roche). Gene expression was analyzed relative to the housekeeping gene elf1α. Primers used for *runx2a, runx2b, sost, bglap, sp7/osterix, postna, acp5a, ctsk, col2a1, htr2b*, and *mgp* are shown in Supplementary Table S5.

### Spinal sections/Col2a1Stainings/Immunohistochemistry

The zebrafish were euthanized in tricaine (MS-222, Sigma-Aldrich) and fixed for 3 days in 4% PFA at 4°C, after the abdomen was opened to ensure proper fixation. The spinal columns were dissected and decalcified with Ethylenediaminetetraacetic acid (EDTA) 10 % for 3 days under agitation at RT. After fixation and decalcification, the zebrafish were embedded in paraffin. Longitudinal sections (5 μm) were obtained and were deparaffinized in xylene and were rehydrated in a graded series of ethanol. The slides were stained with hematoxylin (STATLAB Medical Products, LLC) for 4 min, and washed with alcohol-acid, and were rinsed with tap water. In the blueing step, the slides were soaked in saturated lithium carbonate solution for 10 sec, and then rinsed with tap water. Finally, staining was performed with eosin Y (STATLAB Medical Products, LLC) for 2 min and mounted with Permount™ mounting medium.

For Safranin O/Fast green, after dehydration, the slides were stained with Weigert’s hematoxylin (Sigma-Aldrich) for 4 min, wash under tap water, immersed in alcohol–acid for 5 s, then washed under water. The slides were stained with Fast green 0.02 % for 2 min, washed with acetic water 1% for 20 sec, then stained directly in safranin 0.01 % for 5 min and mounted with Permount™ mounting medium. Images were taken using an AxioZoom V16 (Zeiss) and area and number of nuclei were determined using ImageJ.

Immunofluorescence for Col2a1 was performed for 5 dpf larvae fish which were fixed with 4% PFA overnight. Samples were blocked with 4% BSA in PBT (1% Triton) and samples placed in primary antibody for Col2a1 (1 in 20; Developmental studies hybridoma bank, II-II6B3) at 4°C overnight. Then washed with PBT for several hours and then incubated with secondary antibody Goat-anti-Mouse AlexaFluor 488 (1 in 200) at 4°C overnight.

For 1-year old fish paraffin sections, epitope demasking was performed by digestion with Proteinase K (20 μg/ml). Tissue was blocked with Normal goat serum (NGS) and samples treated with primary antibody for Col2a1 (1 in 10; Developmental studies hybridoma bank, II-II6B3) and secondary antibody Goat-anti-Mouse Alexa fluor 488 (1 in 300). Counter stain was done using 4′,6-diamidino-2-phenylindole (DAPI)-mounting medium. Images were taken using a LSM780 (Zeiss).

### Whole mount *in situ* hybridization

**Whole mount in situ hybridization (**WISH) was performed according to Thisse and Thisse, 2008.^48^ Embryos were treated with 0.003% PTU to inhibit pigment synthesis. Embryos were fixed in 4% PFA overnight and dehydrated in Methanol prior to WISH. Antisense probes for *col2a1a* and *sox9a* were obtained from Dr Kristin Artinger (University of Colorado Anschutz Medical Campus, Aurora, CO, USA). Primers used for *htr2b* probe synthesis were: Forward: 5'- GTGGATTGGCCTTCGCATTG-3'; Reverse: 5'- AAAGCCACCAAAGACCCGTA-3'. Sequences were cloned into the pCRII dual promoter plasmid and transcribed with T7 polymerase.

### TUNEL assay

Whole-mount TUNEL staining to determine apoptosis was performed on 5 dpf larvae. The larvae were initially fixed with 4% PFA, followed by a sequential dehydration and rehydration process using 25%, 50%, and 75% methanol (MeOH) in PBS with 0.1% Tween-20 (PBST). The samples were then washed with PBST for 10 min thrice. Next, the samples were subjected to 75 μg/ml Proteinase K digestion at 30°C for 20 minutes, followed by rinsing with PBS-Tween (0.1%) and re-fixation with 4% PFA for an additional 20 minutes. The samples were then washed for 20 minutes thrice in PBS-Triton (1%) and rinsed once more with PBS prior to the addition of the terminal deoxynucleotidyl transferase (TdT)-mediated dUTP nick-end labeling (TUNEL) reaction mixture for 1 hour at 37°C, as per the manufacturer's instructions (Roche/Sigma-Aldrich). The samples were then washed with PBS, stained with DAPI and mounted on a glass slide in Fluoromount-G™ (Invitrogen). Confocal images were taken using a LSM780 Zeiss microscope and analyzed using ImageJ.

### Bromodeoxyuridine (BrdU) incorporation assays

Zebrafish larvae at 3 dpf were incubated in a 10 mM solution of BrdU (BD Biosciences; Cat# 550891) in fish water at a temperature of 28.5°C for 48 h. All of the larvae were then fixed at 5 dpf in 4% PFA overnight at 4°C and used for fluorescence immunohistochemistry, as described below.

### Whole-mount fluorescence immunohistochemistry

Zebrafish larvae fish (3 or 5 dpf) were fixed with 4% PFA overnight at 4°C. After fixation, samples were washed three times for 10 min with PBST. Samples were then washed in PBS-Triton (1%) for 1 hour and blocked in 10% NGS for 1 h at room temperature. Then the samples were incubated in primary antibodies: BrdU at 1:250 (Abcam, ab152095); Cathepsin K at 1:200 (Proteintech, 11 239-1-AP); pH 3 (Ser10) at 1:200 (Millipore, 06-570); Runx2 at 1:200 (Abcam, AB23981) in blocking solution overnight at 4°C. The following day samples were then washed with PBST for several hours and then incubated with species specific secondary antibodies coupled to Alexa Fluor 488 or 555 (Invitrogen) (1 in 1000) at 4°C overnight. The samples were then washed PBST for 30 min thrice and mounted on a glass slide in Fluoromount-G™ (Invitrogen). Confocal images were taken using a LSM780 Zeiss microscope and analyzed using ImageJ. For proliferation assays, pH 3 or BrdU positive cells were counted in the craniofacial elements including in the region of the Meckel’s cartilage and ceratohyal.

### Tartrate-resistant acid phosphatase staining

Osteoclast activity was assessed using a leukocytes acid phosphatase tartrate-resistant acid phosphatase (TRAP) detection kit (Sigma-Aldrich, 387A) following the manufacturer’s protocol. Zebrafish 9 dpf larvae and adult scales were collected and fixed with 4% PFA. They were washed three times with PBS-Tween (0.1%) for 10 min. Samples were then incubated with the TRAP staining solution for 3 h at 37°C. The samples were then washed in PBST and moved to 80% glycerol in PBST. Analysis of osteoclast activity on scales was performed in ImageJ using the threshold application.

### Alkaline phosphatase staining

Histological alkaline phosphatase activity was detected on 9 dpf larvae and adult fixed in 4% PFA and washed with NTMT buffer (100 mM NaCl, 100 mM Tris–HCL (pH 9.5), 50 Mm MgCl_2_ and 1% Tween-20). Samples were then stained with NBT/BCIP (Sigma-Aldrich) in NTMT buffer. After staining, samples were mounted and imaged using the Zeiss stereomicroscope. Analysis of alkaline phosphatase (ALP) activity on scales was performed in ImageJ using the threshold application.

### Human studies

Lymphoblastoid cell lines (LCLs) from a *CHD7* mutation-positive (c.5050 + 1G > T) individual with CS and its unaffected parents were maintained in RPMI medium as described previously.^49^ Families provided informed consent on studies approved by the respective institutional review board of the Baylor College of Medicine (experimental cohort for this study). Relative transcript levels of *HTR2B* mRNA in LCLs were analyzed by RT-qPCR using the methodology described above. The following primers were used for *HTR2B*: (Forward) 5'- TCTTTTCAACCGCATCCATCA- 3' and (Reverse) 5'-TGCTGTAGCCCGTGAGTTATA-3'. Fold change was calculated according to the 2(−ΔΔCt) method, using *HPRT1* and *RPS1* as housekeeping genes for normalization. All data were expressed as mean fold change ± SD across replicates, relative to control parents set to 1.

### Serotonin receptor Htr2b inhibitor treatment and rescue experiments

Compounds RS127445 or SB204741 were obtained from Sigma-Aldrich (Catalog #: R2533 and S0693, respectively). Stock solutions were made in DMF, which was used as vehicle control for all experiments. Wildtype embryos were treated with the respective Htr2b inhibitor starting at 8hpf until 5dpf. The E3 medium containing the final concentration of the compound was replaced every day, and embryos maintained at 28.5°C until the desired stage.

Rescue experiment was performed using *htr2b* (NM_001044743.1) zebrafish open reading frame was purchased from GenScript. In vitro transcription was done using the T7 message machine kit (Ambion) and 1 nl of *htr2b* mRNA (50 ng/μl) was injected into the 1-cell stage embryos.

### Statistics

All zebrafish larval experiments were performed on at least three replicates (N) and each consisted of a sample size (n) of 5-24 fish. MicroCT experiments were performed on 1-year (n = 8 for vertebrae, n = 5 for skull) and 2-year-old (n = 5) zebrafish. Data are presented as Mean ± SEM. Statistical analysis was carried out using Prism-GraphPad® (GraphPad, San Diego, CA, USA). MicroCT data of *chd7^+/+^* vs *chd7^-/-^* mutants was analyzed using student’s t-test with Welch’s correction and Variance determined by F-test analysis. Further quantifications were either done by Student’s t-test for single comparison or ANOVA test for multiple comparisons. Data are presented as Mean ± SEM, unless otherwise indicated. Significance was determined at ^*^ < 0.05; ^**^ < 0.01, ^***^ < 0.001 and ^****^ < 0.001.

## Results

### Zebrafish *chd7*^*-/-*^ larvae show craniofacial and spinal deformities

**Figure 1:**
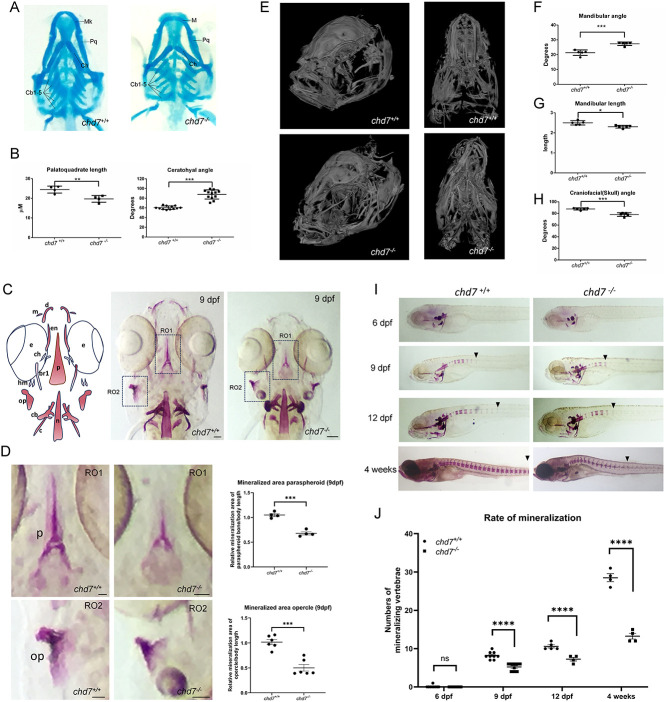
Cartilage and bone defects in *chd7^-/-^* mutant larvae. *(A)* Alcian blue staining of 6 dpf *chd7^+/+^* and *chd7^-/-^* zebrafish larvae, ventral view (Mk: Merkel’s cartilage; Pq: palatoquadrate; Ch: ceratohyal; cb1-5: ceratobranchial cartilages 1-5). (B) Palatoquadrate length (left; n = 4 for both genotypes and ceratohyal angle (right; n = 12 for both genotypes) of *chd7^+/+^* and *chd7^-/-^*. (C) Graphical illustration of craniofacial mineralized tissue in 9 dpf old larvae and Alizarin Red staining of 9 dpf *chd7^+/+^* and *chd7^-/-^* zebrafish larvae, ventral view (m:maxilla; d:dentary; en:entopterygoid; e:eye; p: parasphenoid; ch: ceratohyal; br1: branchiostegal ray1; hm: hyomandibular; op:opercle; cb: ceratobranchial 5; n:notochord). Scale bar = 50 μm. (D) Magnified regions of interest (RO1: paraspheroid and RO2: opercle) of Alizarin Red staining in *C* and quantitative analysis of mineralized area normalized to embryo body length (paraspheroid bone: n = 4 for both genotypes; and opercle: n = 6 for both genotypes). Scale bar = 25 μm. (E) MicroCT analysis of 1-year old adult skulls of *chd7^+/+^* and *chd7^-/-^* in semilateral and ventral view. *(F-H)* Mandibular angle, length and Craniofacial angle of 1-year old fish. *(I)* Alizarin Red staining to show rate of mineralization from 6 dpf to 4 weeks post fertilization *chd7^+/+^* and *chd7^-/-^* zebrafish larvae. Arrows indicate most posterior mineralized centra of vertebrae. *(J)* Quantitative analysis of the rate of mineralization from 6 dpf to 4 weeks post fertilization in *chd7^+/+^* (n = 4-9) and *chd7^-/-^* (n = 4-9) zebrafish larvae. Significance: ^*^P < 0.05; ^**^P < 0.01; ^***^P < 0.001; ^****^P < 0.0001; ns = not significant.

Previously, we generated a *chd7* knockout zebrafish line (*chd7^-/-^*; Allele: *chd7^iaf17^*) using CRISPR/Cas9 that replicates hallmarks of CS,^24^ including craniofacial and skeletal defects (Figure 1A). To gain insights into the role of zebrafish Chd7 in cartilage and bone development, we examined the craniofacial and skeletal anomalies in *chd7^-/-^* mutants in more details. Compared to wild-type (*chd7^+/+^*) larvae, we observed in *chd7^-/-^* larvae at 6 dpf a reduced length of the palatoquadrate (Figure 1B; left panel) and an increased angle of the ceratohyal (Figure 1B; right panel) of the craniofacial cartilage. These defects are accompanied by a marked reduction in mineralization of the craniofacial regions by 9 dpf (Figure 1C), particularly towards the opercle and the paraspheroid bone (Figure 1D). We next assessed the effects of *chd7* deficiency on craniofacial skeleton in adult zebrafish. Morphologically, the skull of *chd7^-/-^* zebrafish is altered in comparison to controls (Figure 1E). In particular, we observed significant alterations in the mandibular angle and length (Figure 1F, G), with the mandibular arch being wider and shorter in *chd7^-/-^* fish. Additionally, we found a marked increase in the craniofacial angle (Figure 1H).

At 5 dpf *chd7^-/-^* larvae were significantly smaller in size than wild-type larvae (Supplementary Figure S1A), consistent with growth retardation as a common feature of CS (2, 3). Additionally, at 9 dpf, we found that 39% (43 of 110 larvae; N = 3) of *chd7^-/-^* larvae exhibited highly variable body curvature phenotypes in both precaudal and caudal regions of the spine (Supplementary Figure S1B). To better understand the etiology of these defects, we next sought to investigate mineralization during larval development using alizarin red staining. We observed a significant reduction in the number of calcified vertebrae in 9 and 12 dpf *chd7^-/-^* larvae compared to control wild-type fish (Figure 1I and J). Notably, *chd7^-/-^* larvae had incomplete calcification of vertebrae towards the posterior region of the spine (Figure 1I). Interestingly, the marked reduction in bone mineralization in *chd7^-/-^* fish persisted in 4-week-old juvenile *chd7^-/-^* zebrafish (Figure 1I and J), suggesting that the decreased mineralization is likely not due to developmental delay at early development stages but rather due to Chd7 deficiency.

### Altered morphology of the Weberian apparatus and abnormal vertebrae in adult *chd7*^*-/-*^ zebrafish

**Figure 2:**
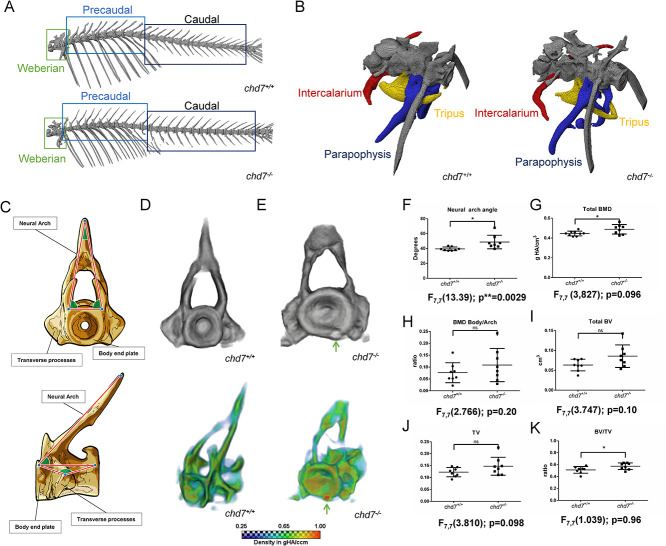
Precaudal vertebrae and Weberian apparatus. (A) Representative microCT overview image of the spinal column from a 1-year old *chd7^+/+^* and a *chd7^-/-^* adult zebrafish. (B) MicroCT Image of the Weberian apparatus from a 1-year old *chd7^+/+^* and a *chd7^-/-^* mutants, indicating structures of intercalarium (red), tripus (yellow) and parapophysis (blue). (C) Sketch diagram of precaudal vertebrae (frontal and lateral) indicating structure and measured angles. (D, E) (top) Individual rendering of precaudal vertebrae, frontal view of tissue over 0.41 g HA/cm^3^ threshold and (bottom) vBMD intensity map (Range blue-red; 0.41 – 1.00 g HA/cm^3^) showing morphological abnormalities and growth zone malformations with highly mineralized inclusions (arrow). (F) Neural arch angle. (G) vBMD of whole vertebrae showing increasing density. (H) Increasing ratio of vBMD arch/vertebrae body. *(I-K)* BV, TV and ratio BV/TV (n = 8/genotype were analyzed). ^*^ denotes *P* < 0.05 and ns = not significant; Student’s t-test.

The structural properties of the spine in both wild-type and *chd7^-/-^* zebrafish were also assessed (Figure 2A). Strikingly, the Weberian apparatus with its supraneurals, intercalarium, tripus and parapophysis, displayed a thinner and smaller morphology in *chd7^-/-^* adults compared to wild-type fish (Figure 2B). We further performed a detailed analysis of volumetric bone mineral density (vBMD), bone volume (BV), total volume (TV), and bone volume fraction (BV/TV) in 1-year-old adult animals. We did not detect any differences in vBMD, BV or TV (Supplementary Table S1). However, as determined by F-test analysis, we observed a significantly greater variance in TV of both the intercalarium and the parapophysis, as well as in BV of the parapophysis in 1-year-old *chd7^-/-^* adults (Supplementary Table S1). The same results were observed in 2-year-old *chd7^-/-^* fish compared to age-matched wild-type controls (Supplementary Table S2). Noteworthy, adult *chd7^-/-^* zebrafish were unchanged in overall body length.

Precaudal and caudal vertebrae structures in *chd7^-/-^* and control 1-year old adult zebrafish were analyzed by assessing key angles of the vertebrae (Figure 2C and Supplementary Figure S2A) and mineralization features. MicroCT analysis of precaudal vertebrae identified altered structures and abnormal patterns of mineralization that were especially pronounced at the arches and transverse processes in *chd7^-/-^* compared to wild-type zebrafish (Figure 2D and E). Structurally, precaudal vertebrae of *chd7^-/-^* mutants had an increased bone volume towards the body end plates of vertebrae and in most cases exhibited major malformations, manifesting as highly mineralized inclusions (Figure 2E, arrows). Analysis of the vertebrae neural arch revealed distortion with a significantly larger rising angle, as well as a larger variance of the angles (Figure 2F). Overall, vertebrae had altered body angle and significant variance of body angle and vertebrae length (Supplementary Table S3).

Precaudal vertebrae in *chd7^-/-^* fish showed significantly higher overall vBMD (Figure 2G), while the distribution of mineralization between the body and arch remained unaffected (Figure 2H). Additionally, analysis of the vertebrae in *chd7^-/-^* zebrafish revealed a larger bone volume fraction (BV/TV) compared to *chd7^+/+^* zebrafish (Figure 2I-K). Noteworthy, in 2-year-old *chd7^-/-^* fish, a depletion of vBMD in the arches and abnormal vertebrae body structure was observed, but the effects seen on total vBMD and BV were not apparent in the few samples that survived until this stage (Supplementary Figure S3 and Supplementary Table S4).

We also determined key structural features (Supplementary Figure S2A) and mineralization characteristics of caudal vertebrae. The *chd7^-/-^* zebrafish caudal vertebrae displayed warped hemal and neural arches and abnormal body structure compared to wild-type zebrafish (Supplementary Figures S2B and S2C). Similar to precaudal vertebrae, the growth zones of the body end plates were enlarged and showed inclusions of highly mineralized matrix (Supplementary Figure S2C). Unlike wild-type zebrafish, *chd7^-/-^* mutants had abnormal vertebrae body structure, which usually exhibited an hourglass shape which resulted in greater variance of measured body angles (Supplementary Table S3).

Similar to the neural arch screened in the precaudal vertebrae, the hemal arch in the caudal vertebrae showed significantly wider arch rising angles (Supplementary Figure S2F), while the neural arch displayed significantly greater variance (Supplementary Figure S2C) in 1-year-old *chd7^-/-^* zebrafish. In contrast to precaudal vertebrae data, the caudal vertebrae showed significantly larger variance of total vBMD and vBMD of both arches and vertebral body (Supplementary Table S3). A significant insufficiency in mineralization of the arches compared to the vertebrae body was also measured in *chd7^-/-^* zebrafish compared to controls (Supplementary Figure S2D and S2E). Further analysis also revealed a significant variance in BV and BV/TV in *chd7^-/-^* zebrafish compared to controls (Supplementary Figure S2H and S2I). While significantly distorted arches were still observed in 2-year-old mutants, the effects on vBMD and BV were not noticed in the few surviving fish that could be screened (Supplementary Figure S4 and Supplementary Table S4).

### Alterations in the expression of cartilage and bone markers in *chd7*^*-/-*^ larvae

**Figure 3:**
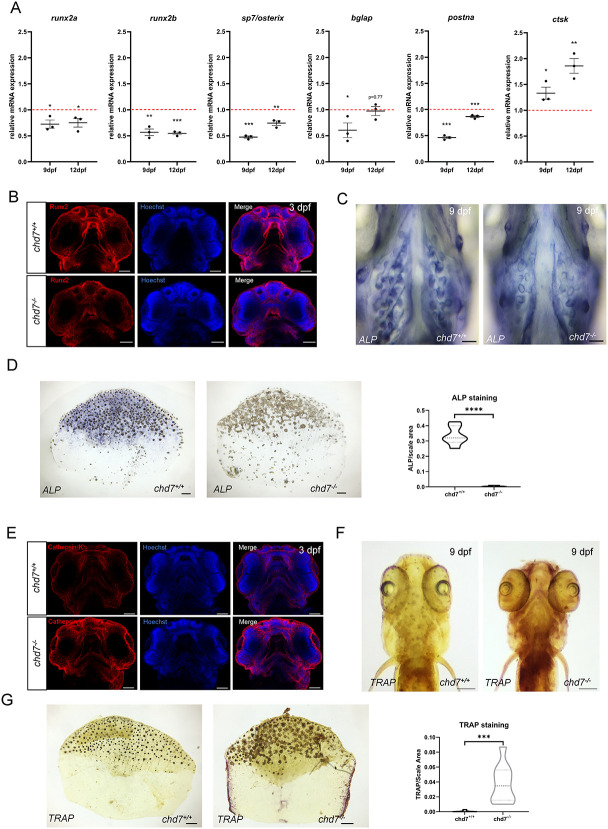
Osteoblasts and Osteoclasts in early development and adults. (A) RT-qPCR of osteoblast (*runx2a, runx2b, sp7/osterix, bglap* and *postna*) and osteoclast related genes (*ctsk*) screened at 9 dpf and 12 dpf in *chd7^+/+^* and *chd7^-/-^*larvae. (B) Immunohistochemistry of osteoblast marker Runx2 (red) and nuclei maker Hoechst (blue) in 3dpf *chd7^+/+^* (n = 5) and *chd7^-/-^* (n = 5) larvae. Scale bar = 60 μm. (C) Alkaline Phosphatase staining (ALP; blue) for osteoblasts in craniofacial region of 9dpf larvae (n = 10 for both genotypes). Scale bar = 200 μm. (D) Alkaline Phosphatase staining (ALP; blue) for osteoblasts in adult scales and quantitative analysis determined by signal/area ratio (n = 6 for *chd7^+/+^* and n = 8 for *chd7^-/-^*). Scale bar = 100 μm. (E) Immunohistochemistry of osteoclast marker cathepsin K (red) and nuclei maker Hoechst (blue) in 3dpf *chd7^+/+^* (n = 5) and *chd7^-/-^* (n = 5) larvae. Scale bar = 60 μm. (F) TRAP staining for osteoclasts in craniofacial region of 9dpf larvae (n = 10 for both genotypes). Scale bar = 100 μm. *(G)* TRAP staining for osteoclasts in adult scales and quantitative analysis determined by signal/area ratio (n = 10 for both genotypes). Scale bar = 100 μm. ^*^p < 0.05; ^**^p < 0.01 ^***^p < 0.001; ^***^p < 0.0001.

To gain insights in the molecular mechanisms underlying the craniofacial and skeletal defects in *chd7^-/-^* fish, we examined osteogenesis and chondrogenesis as potential contributors. In *chd7^-/-^* larvae compared to wild-type larvae, we observed a significant downregulation of osteoblast differentiation markers, *runx2a*, *runx2b*, *sp7/osterix, bglap*, and *postna* in *9* dpf *chd7^-/-^* larvae (Figure 3A). We also detected a significant upregulation of the osteoclast marker *ctsk* in *chd7^-/-^* larvae (Figure 3A). Expression patterns of *runx2a*, *runx2b*, *sp7/osterix, postna*, and *ctsk* remain dysregulated in *chd7^-/-^* larvae even as they age (Figure 3A, 12 dpf). Additionally, the *chd7^-/-^* larvae had significantly lower levels of the osteocyte marker and Wnt signaling inhibitor, *sost,* as well as other genes known to regulate mineralization such as *acp5a*, while *mgp* remained unchanged compared to wild-type larvae (Supplementary Figure S5A).

We next sought to determine the basis of the osteogenic defects in *chd7^-/-^* mutants by assessing the progression of osteogenesis at various developmental stages. At 3 dpf, we observed reduced levels of the Runx2 protein in *chd7^-/-^* mutants compared to controls (Figure 3B). We then examined osteoblastic activity in 9 dpf larvae and in the adult skeleton using ALP, which is highly expressed in primary osteoblasts. At adult stage, the zebrafish elasmoid scales are translucent mineralized structure of the dermal skeleton and represent a useful model for analyzing osteoblast and osteoclast behaviour during bone formation/remodeling and mineralization. We found a marked reduction in ALP staining in 9 dpf *chd7^-/-^* larvae (Figure 3C) and scales (Figure 3D) compared to controls, suggesting alteration in osteogenic differentiation upon loss of function of Chd7.

We then analyzed osteoclast activity using either the osteoclast marker, cathepsin K, or TRAP staining. At 3 dpf, we observed a higher expression of cathepsin K in *chd7^-/-^* mutants compared to *chd7^+/+^*(Figure 3E)*.* We also found a significant increase in TRAP staining at 9 dpf in *chd7^-/-^* mutants (Figure 3F) and *chd7^-/-^* scales (Figure 3G), suggesting higher osteoclast activity upon Chd7-deficiency.

**Figure 4:**
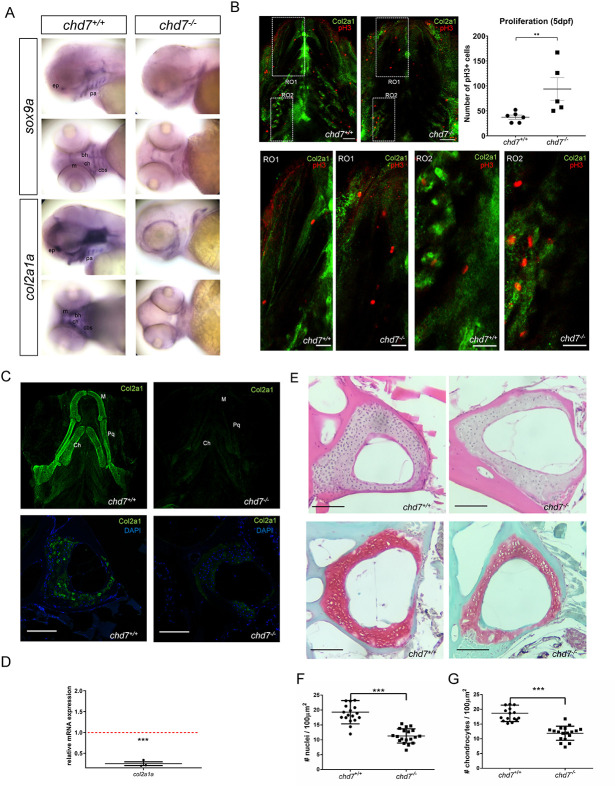
ECM Collagen2a1 deficiency in *chd7^-/-^* larvae and adults. (A) WISH of *sox9a* and *col2a1a* in craniofacial regions of *chd7^+/+^* and *chd7^-/-^* 3dpf larvae, lateral and ventral view (ep: ethmoid plate; pa: palatoquadrate; m: Meckel’s cartilage; ch: ceratohyal; bh: basihyal; cbs: ceratobranchials). (B) Immunohistochemistry for proliferation marker pH 3 (red) and Col2a1 (green) in the craniofacial region of 5dpf *chd7^+/+^*(n = 6) and *chd7^-/-^* (n = 5) larvae (top; scale bar = 40 μm) and magnified regions of interest (RO1: Region of the Meckel’s cartilage and ceratohyal; RO2: ceratobranchials) (bottom panels; scale bar = 20 μm.) with quantitative analysis of pH 3 positive cells of the craniofacial region (top right panel). (C) Immunofluorescence of Col2a1 in 5 dpf *chd7^+/+^*(n = 9) and *chd7^-/-^* (n = 8) larvae, ventral view of craniofacial cartilage (top panel; De: dentary; Pq: palatoquadrate; Ch: ceratohyal). Immunofluorescence of Col2a1 in precaudal vertebrae sections of *chd7^+/+^* and *chd7^-/-^* mutants with Col2a1 in green and DAPI in blue (bottom panel). (D) RT-qPCR of *col2a1a* at 9dpf, relative fold change in comparison to *chd7^+/+^* (red dotted line). *(E)* H&E staining of precaudal vertebrae section of *chd7^+/+^* and *chd7^-/-^* mutants (top). Safranin O/fast green staining of Weberian and precaudal vertebrae sections of *chd7^+/+^* and *chd7^-/-^* mutants showing cartilage in red (bottom). (F,G) Total nuclei count in Weberian and precaudal vertebral cartilage detected in H&E staining per 100μm^2^ in *chd7^+/+^* (N = 4, n = 17) and *chd7^-/-^* (N = 4, n = 18) and Chondrocyte nuclei count in precaudal vertebral cartilage detected in Safranin O/fast green staining per 100μm^2^ *chd7^+/+^* (N = 4, n = 18) and *chd7^-/-^* (N = 4, n = 19). Scale bar represents 100 μm. Significance: ^**^P < 0.01 ^***^P < 0.001.

To determine whether Chd7 is also involved in regulating cartilage development, we examined the expression of genes involved in chondrogenesis by whole-mount in situ hybridization. In 72 hpf *chd7^+/+^* zebrafish, *sox9a* and *col2a1a* are expressed within the forming Meckel’s cartilage, the basihyal cartilage, the ceratohyal cartilage and the ceratobranchial cartilages 1-5 (Figure 4A). The expression of chondrocyte markers *sox9a* and *col2a1a* were markedly reduced in *chd7^-/-^* larvae at 72 hpf, particularly in the Meckel’s cartilage and the ceratohyal was significantly reduced in *chd7^-/-^* larvae (Figure 4A). Strikingly, in *chd7^-/-^* larvae the basihyal cartilage and the ceratobranchial cartilages had no expression of *sox9a* or *col2a1a* (Figure 4A). We next assessed cell proliferation in the craniofacial skeleton in 5 dpf *chd7^-/-^* mutant and control larvae using the proliferation marker phospho-Histone H3 (pH 3) (Figure 4B). We observed a significant increase in proliferating cells in the craniofacial elements (Figure 4B), particularly in the region of the Meckel’s cartilage, ceratohyal (Figure 4B; RO1, bottom panels) and ceratobranchials (Figure 4B; RO2, bottom panels) of *chd*7^-/-^ larvae compared to controls. BrdU incorporation assay also demonstrated a higher number of BrdU+ cells in the head region of the pharyngeal in *chd7^-/-^* mutants in contrast to that of *chd7^+/+^* fish at 5 dpf (Supplementary Figure S5B). Altogether, our data suggest that chondrogenesis is altered in *chd7^-/-^* mutants.

Next, we asked whether apoptosis is altered in *chd7^-/-^* mutants. Using a TUNEL assay, we found no change in the number of apoptotic cells in the head skeleton of 5 dpf *chd*7^-/-^ larvae compared to controls (Supplementary Figure S5C).

### *Col2a1* of the ECM is depleted in *chd7*^*-/-*^ zebrafish

To perform a more complete analysis of the skeletal phenotype, we also examined the extracellular matrix (ECM) of bone structures in *chd7^-/-^* zebrafish. Antibody staining showed that all craniofacial structures are already greatly depleted of the key Col2a1a protein by 5 dpf (Figure 4C, top). In adult *chd7^-/-^* zebrafish, immunofluorescence showed a striking depletion of Col2a1 expression in the cartilage of vertebrae seen in the Weberian and precaudal vertebrae (Figure 4C, bottom). This pattern was effectively reproduced in all regions of the spine of *chd7^-/-^* zebrafish. In line with the WISH and immunostaining experiments, gene expression analysis at 9 dpf revealed a significant downregulation of *col2a1a* (Figure 4D).

We further tested for cell nuclei and chondrocytes in vertebral cartilage. Hematoxylin–Eosin (HE) staining of the Weberian apparatus and precaudal vertebrae sections showed a significant reduction in the number of nuclei measured in the vertebral cartilage of 1-year-old *chd7^-/-^* compared to *chd7^+/+^* zebrafish (Figure 4E, top and 4F). Complementary analysis using Safranin/Fast Green O staining, revealed a significant reduction in the number of chondrocytes in the vertebral cartilage of *chd7^-/-^* zebrafish in line with the H&E staining (Figure 4E, bottom and 4G). Altogether, the findings indicate that loss of *chd7* leads to an altered cartilage composition in addition to the observed bone deformations.

### *Htr2b* downregulation in *chd7*^*-/-*^ zebrafish leads to craniofacial defects

**Figure 5:**
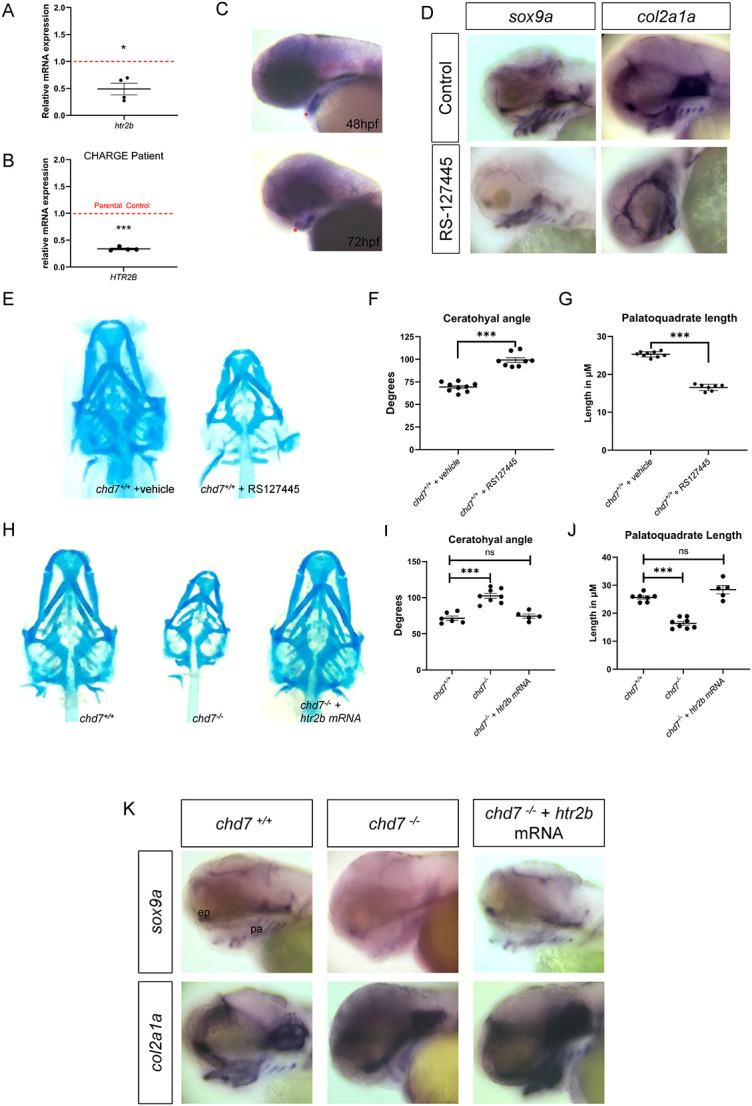
Role of Htr2b in craniofacial development. (A) RT-qPCR of *htr2b* at 3dpf, relative fold change in comparison to *chd7^+/+^*(N = 4). (B) RT-qPCR of *HTR2B* mRNA in a LCL from a CS patient in comparison to parental controls (N = 4). (C) WISH of *htr2b* in 48 hpf and 72hpf zebrafish, lateral view. Red asterisk indicates expression in craniofacial structures. (D) WISH of *sox9a* and *col2a1a* in craniofacial regions of control or *Htr2b* inhibitor treated larvae, lateral view. (E) Alcian blue staining of 6 dpf larvae treated with vehicle (DMF) or Htr2b inhibitor RS127445. (F, G) Statistical analysis of palatoquadrate length and ceratohyal angle in vehicle or inhibitor treated larvae (n = 9 for *chd7^+/+^* and n = 8 for *chd7^-/-^*). (H) Alcian blue staining of 6 dpf larvae of wildtype, *chd7^-/-^* and *chd7^-/-^* injected with *htr2b* mRNA. (I,J) Statistical analysis of palatoquadrate length and ceratohyal angle *chd7^-/-^*(n = 6-7), *chd7^-/-^* (n = 8) and *chd7^-/-^* injected with *htr2b* mRNA(n = 5). (K) WISH of *sox9a* and *col2a1a* in craniofacial regions of 72hpf wildtype, *chd7^-/-^* and *chd7^-/-^* injected with *htr2b* mRNA larvae, lateral view (ep: ethmoid plate and pa: palatoquadrate). Significance of Student’s t-test or ANOVA with: ^*^P < 0.05; ^**^P < 0.01; ^***^P < 0.001; ns = not significant.

We next sought to understand the mechanisms underlying craniofacial defects upon loss of function of CHD7. The serotonin receptor HTR2B (5-hydroxytryptamine receptor 2b) has unexpectedly been shown to play a role in craniofacial development.^50^ Through RNA-sequencing (RNA-seq), we recently identified a severe downregulation of *htr2b* transcription (Fold-change: -2.96, *P*-value: 1.68 × 10^-12^; GSE139623) upon loss of function of *chd7* in zebrafish.^24^ To validate our RNA-seq data, we performed qPCR analysis on *chd7^-/-^* and *chd7^+/+^* zebrafish larvae. We found a significant reduction in *htr2b* expression in *chd7^-/-^* zebrafish larvae (Figure 5A). Furthermore, at 48 and 72 hpf, we showed that *htr2b* is highly expressed in the jaw and brain of zebrafish larvae (Figure 5C); structures that are most notably affected in CS. To validate that this finding was clinically relevant, we examined *HTR2B* expression in lymphoblastoid cell lines (LCLs) derived from a *CHD7* mutation-positive child and its unaffected parents. LCLs are especially useful for analysing molecular mechanisms relevant for CS.^24^^,^^49^ RT-qPCR analysis showed that *HTR2B* gene expression was robustly decreased in the *CHD7* mutation-positive LCL compared to parental control LCLs (Figure 5B).

We next sought to examine the effects of reduced Htr2b signalling on zebrafish craniofacial development. To determine whether Htr2b is involved in regulating cartilage development, we examined the expression of the chondrocyte markers *sox9a* and *col2a1a* in zebrafish treated with or without Htr2b inhibitors (Figure 5D). Treatment with Htr2b inhibitors results in a marked reduction in the expression of *sox9a* and *col2a1a* in zebrafish. Interestingly, pharmacological inhibition of Htr2b with the inhibitor RS-127445 strikingly mimics the morphological features observed in the *chd7^-/-^* zebrafish larvae (Figure 5E), including reduction in the length of the palatoquadrate and increased ceratohyal angle (Figure 5F and G). Inhibition with another Htr2b inhibitor (SB204741) validated these results (Supplementary Figure S6).

Since the expression of *htr2b* is downregulated in *chd7^-/-^* zebrafish, we reasoned that *htr2b* overexpression may rescue cartilage defects induced by loss-of-function of *chd7*, if Chd7 regulates Htr2b. Overexpression of *htr2b* mRNA rescued the craniofacial phenotype in *chd7^-/-^* larvae and fully recovered the reduction of palatoquadrate length and ceratohyal angle (Figure 5H-J). Importantly, overexpression of *htr2b* mRNA in in *chd7^-/-^* zebrafish larvae rescued the expression of the chondrogenesis markers *sox9a* and *col2a1a* (Figure 5K). Noteworthy, the reduced mineralization at 9 dpf was not rescued by overexpression of *htr2b* mRNA in in *chd7^-/-^* zebrafish larvae (Supplementary Figure S7). Altogether, these data suggest that reduced Htr2b signalling during zebrafish development disrupts chondrogenesis under the control of Chd7.

## Discussion

The present study provides a detailed description of bone and craniofacial phenotypes upon loss-of-function of Chd7 in zebrafish and novel mechanistic insights to support a crucial role for Chd7 in craniofacial cartilage development via controlling Htr2b expression. We characterized the morphological, cellular, and molecular defects in the craniofacial and spinal vertebrae in zebrafish *chd7^-/-^* mutant larvae and adults. A delay in bone mineralization was found in larval *chd7^-/-^* fish accompanied by dysregulation of osteogenesis genes. We observed defects in chondrogenesis upon loss of function *chd7* in zebrafish due to downregulation of *htrb2b* expression.

We demonstrated that *chd7^-/-^* zebrafish display craniofacial and skeletal anomalies reminiscent of those observed in CS patients.^9-11^ For example, CS patients commonly present with square face, broad nasal bridge, small mouth and facial asymmetry.^8^ Abnormal curvature of the spine (scoliosis or kyphosis) occurs in many CS patients and has been attributed to poor muscular development. Low bone mineral status has been reported in adolescent idiopathic scoliosis.^51^ We found impaired early bone mineralization of vertebrae and a significant reduction in osteogenesis markers including *sp7/osterix* in larval *chd7^-/-^* zebrafish. Misregulation or loss of osteogenesis-related genes, such as *sp7/osterix* has been directly linked to poor bone mineralization in zebrafish and humans.^52-54^ Additionally, the increased osteoclast and decreased osteoblast activity likely contribute to the reduced bone mineralization in larval *chd7^-/-^* mutants. It has been shown that BMD is significantly reduced in CS patients.^15^ Perhaps, poor bone quality may be an etiological factor of spinal deformity in people with CS. Early analysis of bone density and spinal development has been suggested by CS guidelines.^55^^,^^56^ Additional work is needed to examine bone quality as a possible etiological factor of spine deformities in individuals with CS.

Our analysis of volumetric bone mineral density (vBMD) and other parameters (e.g. BV, TV) in adult *chd7^-/-^* fish revealed large variance in density of vertebrae. This observation is in line with CS patients showing varying severity in spinal deformities.^11^^,^^57^ Additionally, in most *chd7^-/-^* mutant fish we observed highly mineralized inclusions, most commonly, localized towards the growth zone of the vertebrae. Future studies are required to more closely examine these previously unidentified inclusions. It remains unknown if these mineralization inclusions in the fish vertebrae are also present in vertebrae of people with CS and whether they alter the structural integrity of the spine in people with CS. It is likely that detection of these inclusions would be missed during dual-energy x-ray absorptiometry screens of people with CS when assessing aBMD. Higher resolution *in vivo* quantitative computed tomography or postmortem assessment of vertebrae would likely be required to identify these mineralization defects. Noteworthy, during larval stages, we observed a reduced bone mineralization in *chd7^-/-^* mutants but we did not observe an overall lower BMD in the adult *chd7^-/-^*. It is plausible that either as the fish grows the delay in mineralization is caught up or that *chd7^-/-^* fish with lower BMD die prematurely. Indeed, very few *chd7^-/-^* zebrafish survive past 12 dpf to juvenile and adulthood stages.^24^

Notable, at 5dpf, while no apoptosis was detected, a significant increase in pH 3 positive cells was observed in regions of craniofacial cartilage in *chd7^-/-^* larvae. As *chd7^-/-^* mutants had a marked reduction in the expression of Col2a1, we could not perform Col2a1 and pH 3 colocalization analysis in our proliferation assays. It is possible that the pH 3 positive cells are chondroprogenitors rather than chondrocytes and that a failure in differentiation of progenitor cells into chondrocytes occurs upon chd7 deficiency. However, further studies are warranted to better understand chondrogenesis defects in *chd7^-/-^* fish.

A striking observation in *chd7^-/-^* zebrafish is the marked decrease in the collagen component Col2a1 in the craniofacial and vertebral cartilage. Col2a1 is required in the ECM of various cell types including osteoblasts, chondrocytes, external ligament connective tissue cells, as well as notochord basal cells and expressed in the corresponding tissues.^58^^,^^59^ Additionally, *col2a1a* expression is observed throughout ossification of the notochord in zebrafish.^60^ Reduction in this collagen matrix in the notochord can affect proper calcification and is known to cause spondyloepiphyseal dysplasia congenita, which affects skeletal and spine development.^61^ Interestingly, *sedc* mice, which are deficient in *Col2a1*, show enlargement and malformations in the growth plates of vertebrae and degeneration of cartilage, similar to our study in zebrafish.^62^ *Col2a1a* is expressed in osteoblasts and chondrocytes of teleosts and regulated by *sox9*, which in turn is known to be regulated by *chd7.*^17^^,^^60^^,^^63-65^ Sox9 is also well known to be required in craniofacial development, as its related factor Sox10, both of which have been connected to the craniofacial phenotype in CS.^18^ Our study shows loss of function of *chd7* disrupt chondrogenesis and bone ossification in zebrafish. Consistent with our findings, craniofacial defects and reduced c*ol2a1* expression have also been observed upon knockdown of *chd7* in *xenopus*.^17^^,^^66^ A clinical case study of an individual with CS from birth to adulthood (33-year-old) reported the development of osteoporosis in this person.^67^ Therefore, it may be important to monitor bone density in people with CS.

We found a significant reduction in the expression of serotonin receptor *htr2b* in *chd7^-/-^* zebrafish and in cells from people with CS. Pharmacological inhibition of Htr2b affects chondrogenesis in zebrafish similar to what was observed in *chd7^-/-^* fish, suggesting that Chd7 regulates cartilage development via controlling *htr2b* expression. The lack of a specific antibody recognizing zebrafish Chd7 precluded us from performing chromatin immunoprecipitation-PCR (ChIP-PCR) analysis to assess whether *htr2b* is a direct target or not of Chd7 in zebrafish. Analysis of the ChIP-sequencing (ChIP-seq) datasets from the ENCODE Transcription Factor Targets project,^68^ revealed that *HTR2B* is not a direct target of CHD7 in murine and human cell lines. CHD7 interacts and regulates transcriptional activity of *RUNX1* which has been shown to interact with regulatory elements from the *HTR2B* gene.^69^ Knockdown of *runx1* in zebrafish disrupts craniofacial cartilage development in zebrafish. It is thus plausible that CHD7-RUNX1 axis regulates chondrogenesis via controlling HTR2B expression. Subsequent studies are needed to investigate the role of the CHD7-RUNX1 axis in cartilage development. Interestingly, Htr2b signaling is essential for craniofacial development in *xenopus*.^50^ There is also clear evidence in the literature to support a role of serotonin during mammalian craniofacial development.^70^ Additionally, serotonin and its receptor HTR2B have been implicated in NCC migration^50^^,^^71^. The novelty of our findings is that we demonstrated that craniofacial malformations in CS patients may be attributed to dysregulation of HTR2B functioning upon mutations in *CHD7*.

In *chd7^-/-^* zebrafish the expression of *col2a1a* is severely depleted. It is worth noting that pharmacological inhibition of Htr2b in zebrafish does not completely deplete *col2a1a* expression, but replicates cartilage defects observed in *chd7^-/-^* larvae. It is possible that other Chd7 target genes in addition of *htr2b* may impact the expression of *col2a1*. Chd7 has recently been reported to regulate the TGF-β signaling pathway^66^ which in turn is well-known to stimulate the expression of ECM components, such as fibronectin and collagen.^72^ Interestingly, serotonin and its receptor HTR2B control expression of TGF-β genes and activates TGF-β signaling.^73^^,^^74^ Future studies are needed to determine whether Col2a1 expression is regulated by TGF-β/Serotonin signaling.

In summary, our study is a thorough analysis of skeletal abnormalities linked to *chd7* deficiency in a zebrafish model of CS. Our work demonstrates that Chd7 regulates the expression of chondrogenesis and osteogenesis markers. Importantly, we identify a key molecular pathway implicating regulation of *htr2b* by Chd7 in skeletal development. Our work provides the basis for investigating the therapeutic potential of targeting HTR2B in CS for treating craniofacial anomalies.

## Supplementary Material

S1_Supplemental_Files_JBMR2023_2024_zjae024

## Data Availability

The data that support the findings of this study are available from the corresponding author upon reasonable request.
