# Supplementary material for: CHD7 regulates craniofacial cartilage development via controlling HTR2B expression
Source: J Bone Miner Res. 2024 Feb 4;39(4):498–512. doi: 10.1093/jbmr/zjae024 (PMC11262153; doi:10.1093/jbmr/zjae024)
Supplement: S1_Supplemental_Files_JBMR2023_2024_zjae024 [file S1_Supplemental_Files_JBMR2023_2024_zjae024.pdf]

Supplemental Figures

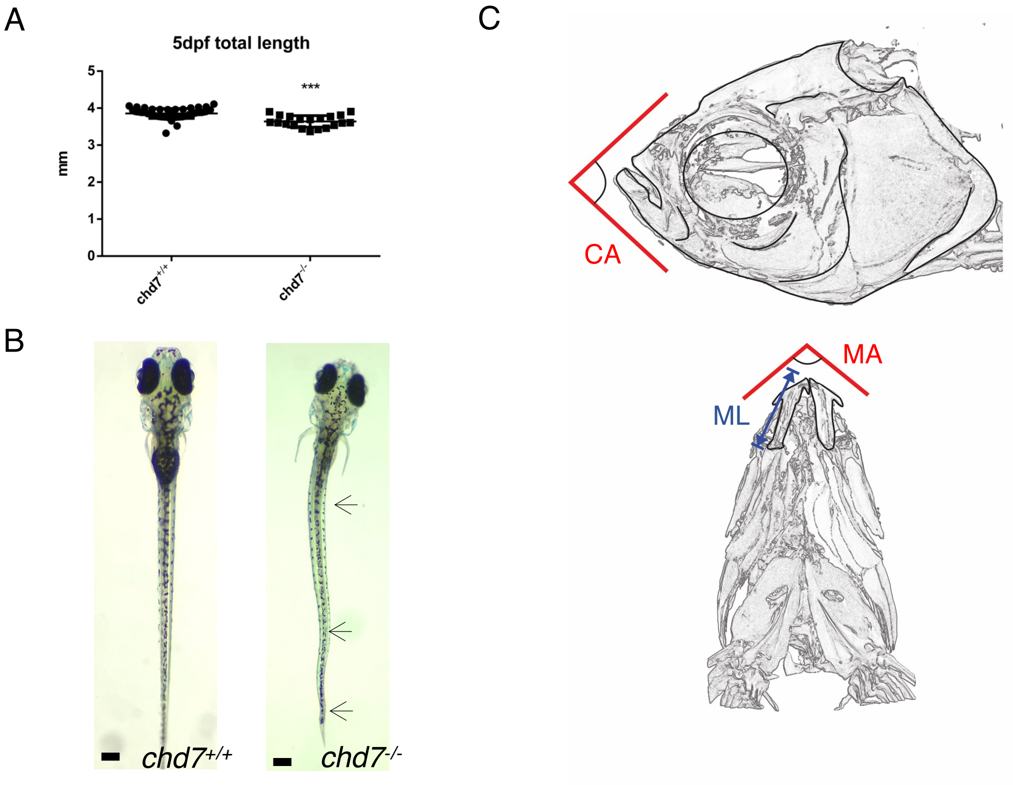

Fig S1. Body morphological analysis in *chd7* $^{-/-}$ mutants and cranial parameters. *(A)* Total length of 5dpf larvae. *(B)* Dorsal view of *chd7* $^{+/+}$ and *chd7* $^{-/-}$ larvae at 9 dpf showing body curvature deformities in 43 out of 110 larvae of 3 separate clutches screened. Deformities were present at both precaudal and caudal region (arrows). *(C)* Graphical representation of measured cranial parameters of adult zebrafish (CA: craniofacial angle; ML: mandibular length; MA: mandibular angle). Scale bar: 200 µm. ***p<0.001; Student's t-test.

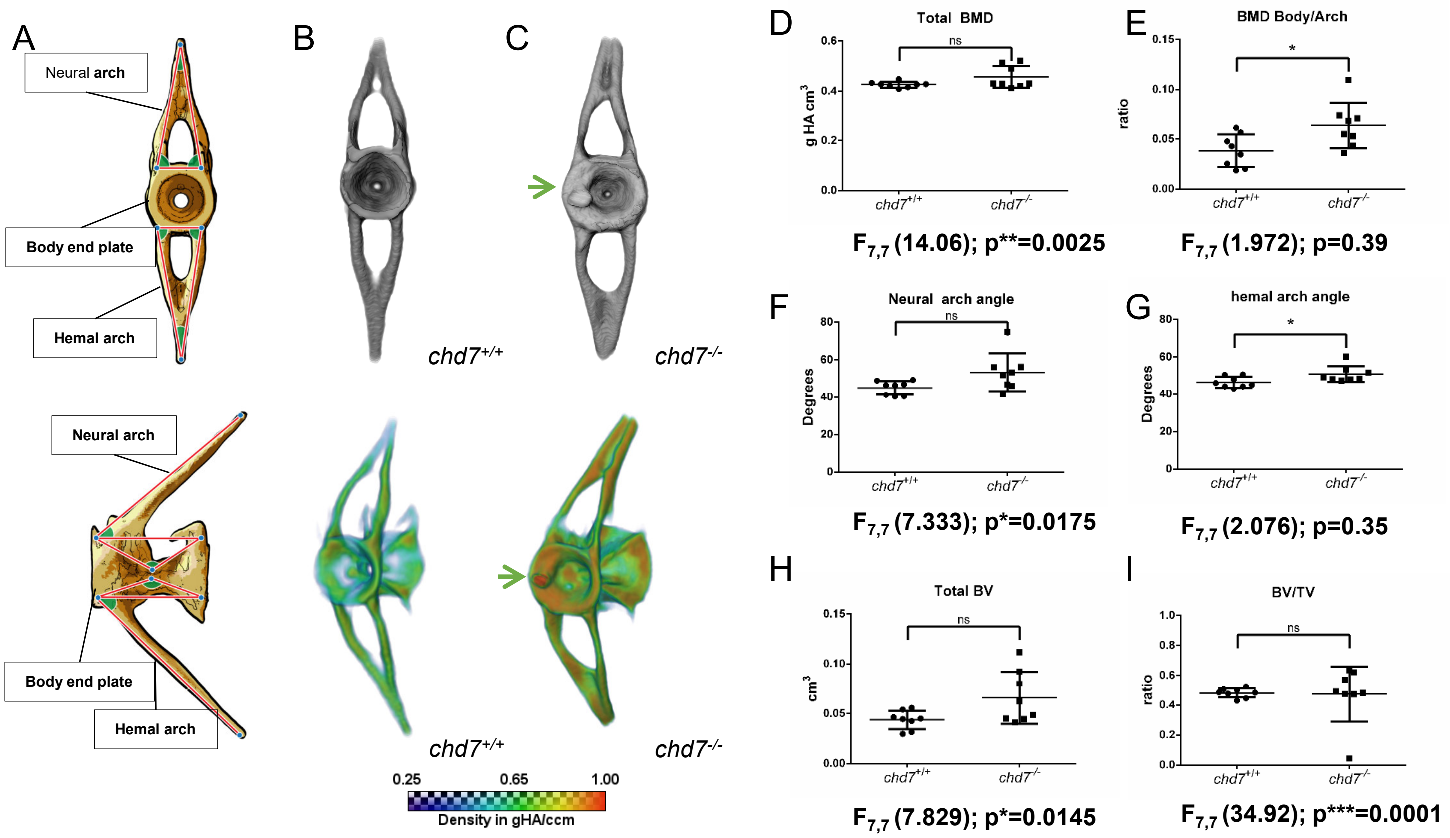

Fig S2. Caudal vertebrae in *chd7*$^{+/+}$ and *chd7*$^{-/-}$ fish.

(A) Sketch diagram of precaudal vertebrae (frontal and lateral) indicating structure and measured angles. *(B, C)* (top) Individual rendering of precaudal vertebrae, frontal view of tissue over 0.41 g HA/cm3 threshold and (bottom) vBMD intensity map (Range blue-red; 0.41 – 1.00 g HA/cm3) showing morphological abnormalities and growth zone malformations with highly mineralized inclusions (arrow). (*D)* vBMD of whole vertebrae. € increasing ratio of vBMD arch/vertebrae body. *(F)* Neural arch angle. *(G)* Hemal arch angle. *(H, I)* BV and BV/TV ratio (N=8/genotype were analyzed). Significance of Student's t-test with Welch's correction is included in the graphs with: ns=not significant; *p<0.05.

Supplemental Figures

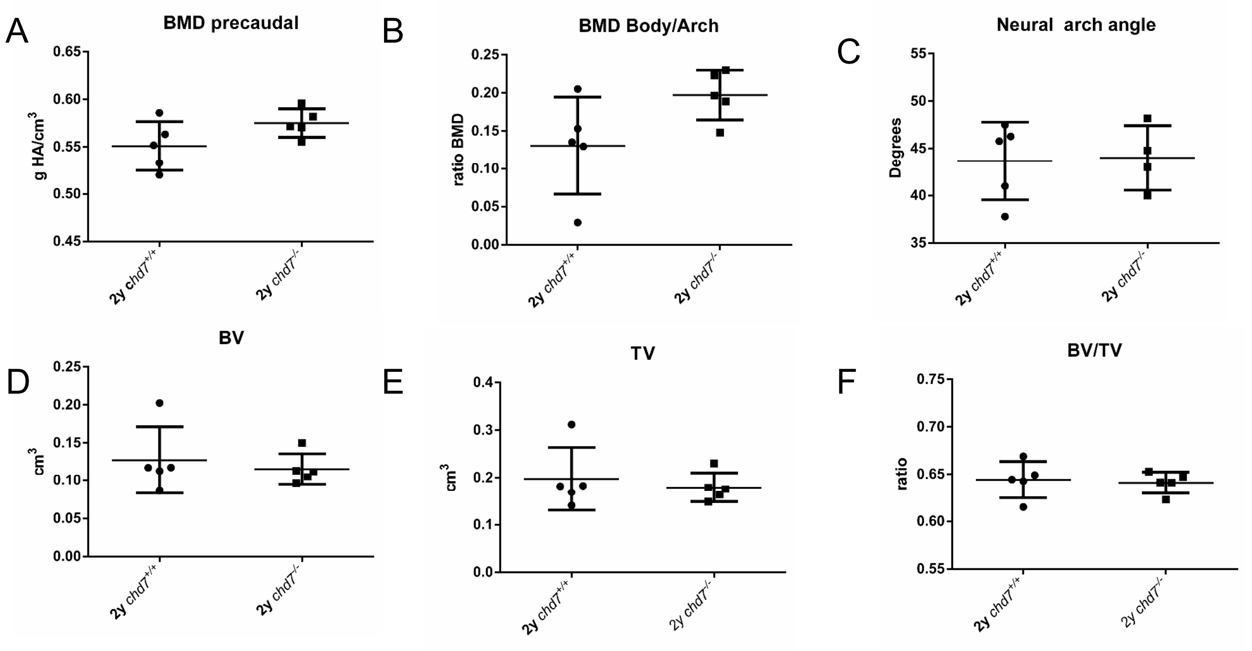

Fig S3. Analysis of key mineralization factors of precaudal vertebrae in 2-year-old *chd7* mutants. Quantitative analysis of *(A)*BMD of whole vertebrae*, (B)* ratio of BMD arch/vertebrae body*, (C)* neural arch angle, *(D)* BV, *(E)* TV and *(F)* ratio BV/TV.

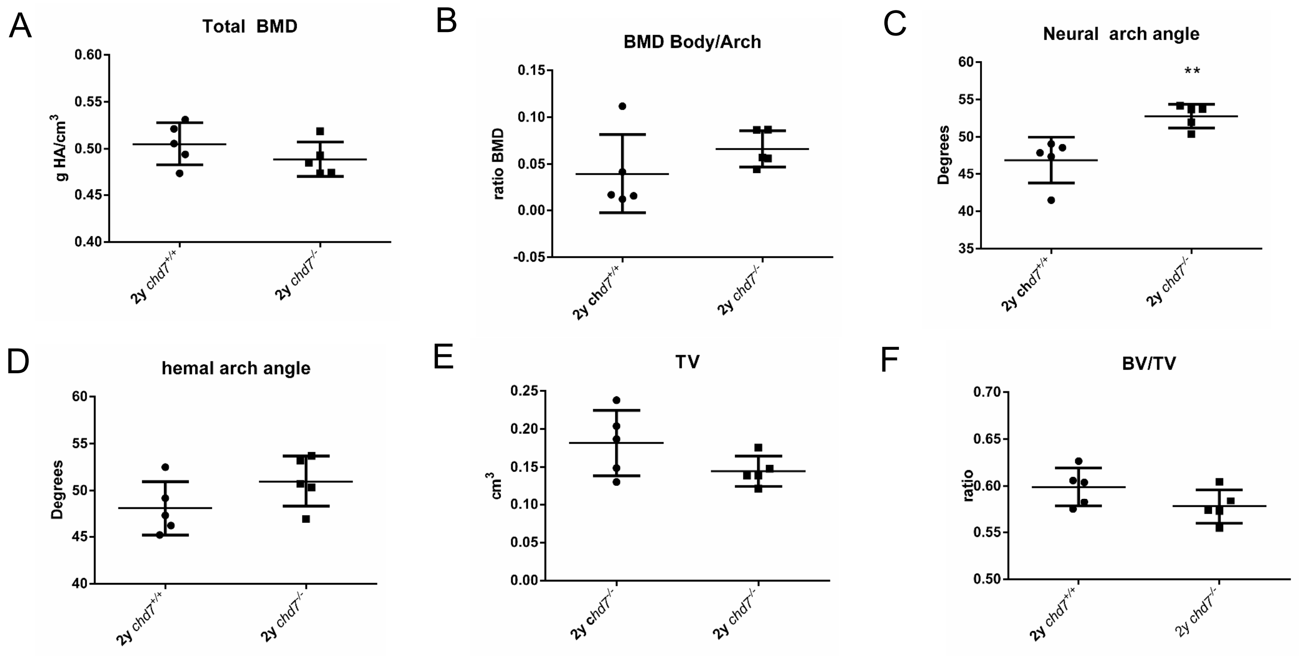

Fig S4. Analysis of key mineralization factors of caudal vertebrae in 2-year-old *chd7* mutants. Quantitative analysis of *(A)* BMD of whole vertebrae, *(B)* ratio of BMD arch/vertebrae body, *(C)* significant increase in neural arch angle, *(D)* hemal arch angle, *(E)* TV and *(F)* ratio BV/TV. ** denotes $p<0.01$.

Supplemental Figures

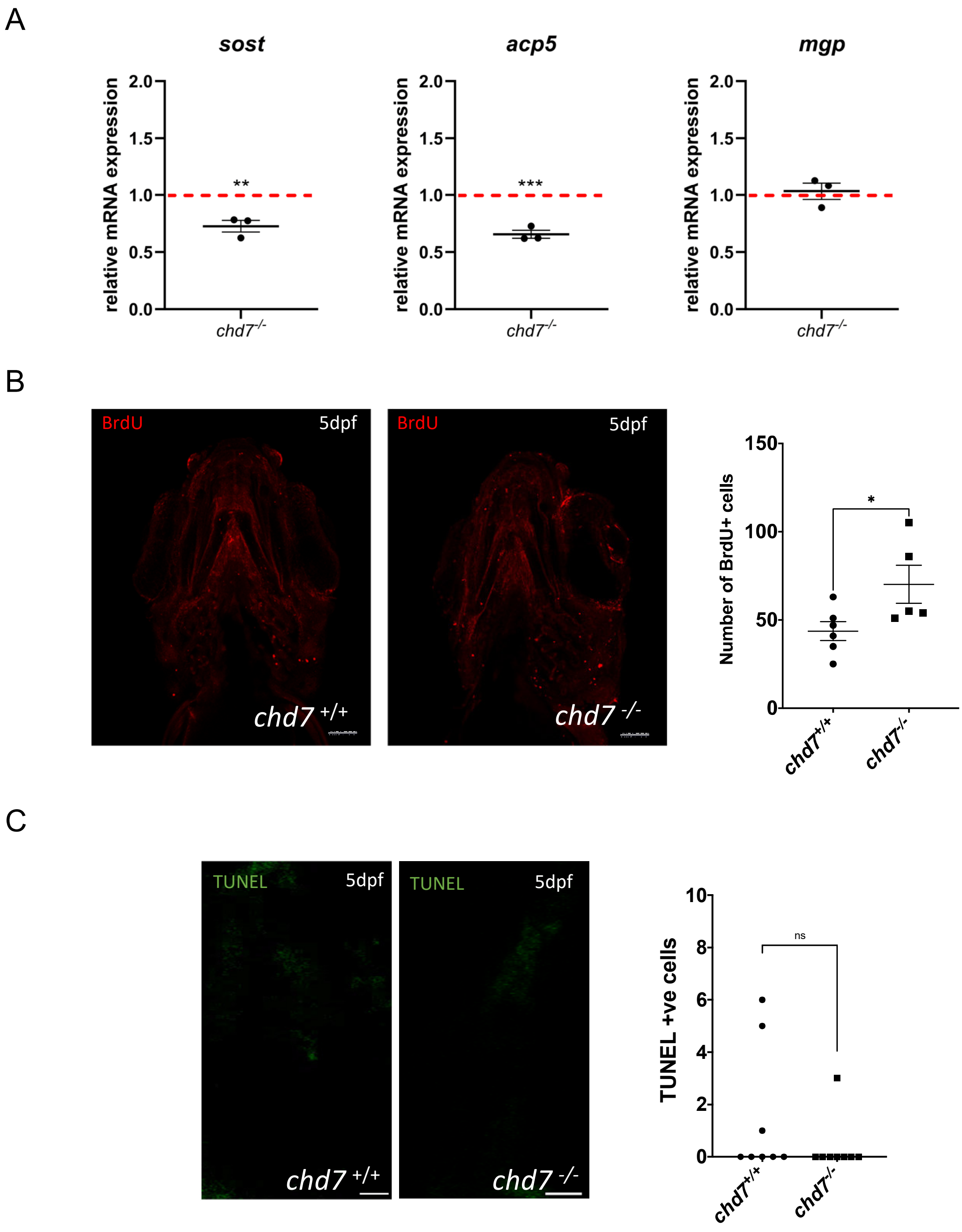

Fig S5. Osteogenesis markers, proliferation and apoptosis in *chd7*$^{-/-}$ mutants. *(A)* RT-qPCR of *wnt* and Osteogenesis markers *sost*, *acp5a* and *mgp* at 9dpf (N=3). *(B)* BrdU staining (red) for proliferation screening and statistical analysis in *chd7*$^{+/+}$ (n=6) and *chd7*$^{-/-}$ (n=5) larvae at 5dpf (ventral view). Scale bar= 40 µm. *(C)* TUNEL staining (green) for apoptosis screening and statistical analysis in *chd7*$^{+/+}$ (n=8) and *chd7*$^{-/-}$ (n=8) larvae at 5dpf (ventral view). Scale bar= 30 µm. *p<0.05, **p<0.01, ***p<0.001 and ns= not significant.

Supplemental Figures

S1 Table. Statistical analysis of mineralization of the Weberian apparatus in 1-year-old zebrafish

	CHD7 $^{+/+}$ 1-year-old			CHD7 $^{-/-}$ 1-year-old				
Weberian structures	n = 8			n = 8			*p(t-test)*	*p(F-test)*
**Intercalarium.TV [mm³]**	0.046	±	0.009	0.056	±	0.020	0.210	0.048
**Intercalarium.BV [mm³]**	0.028	±	0.007	0.035	±	0.014	0.261	0.096
**Tripus.TV [mm³]**	0.085	±	0.025	0.085	±	0.048	0.987	0.101
**Tripus.BV [mm³]**	0.051	±	0.024	0.047	±	0.038	0.823	0.244
**Parapophysis.TV [mm³]**	0.199	±	0.037	0.213	±	0.100	0.710	0.018
**Parapophysis.BV [mm³]**	0.136	±	0.036	0.145	±	0.081	0.777	0.048

S2 Table. Statistical analysis of mineralization of the Weberian apparatus in 2-year-old old zebrafish

	CHD7 $^{+/+}$ 2-year-old			CHD7 $^{-/-}$ 2-year-old			
Weberian structures	n = 5			n = 5			*p(t-test)*
**Intercalarium.BMD [gHA/cm³]**	0.531	±	0.031	0.531	±	0.024	0.979
**Intercalarium.TV [mm³]**	0.076	±	0.032	0.063	±	0.008	0.416
**Intercalarium.BV [mm³]**	0.049	±	0.022	0.040	±	0.006	0.445
**Intercalarium.BV/TV**	0.637	±	0.032	0.641	±	0.028	0.837
**Tripus.BMD [gHA/cm³]**	0.498	±	0.043	0.486	±	0.069	0.751
**Tripus.TV [mm³]**	0.115	±	0.046	0.113	±	0.047	0.963
**Tripus.BV [mm³]**	0.073	±	0.036	0.070	±	0.039	0.885
**Tripus.BV/TV**	0.624	±	0.048	0.592	±	0.075	0.449
**Parapophysis.BMD [gHA/cm³]**	0.592	±	0.036	0.590	±	0.014	0.949
**Parapophysis.TV [mm³]**	0.334	±	0.185	0.220	±	0.028	0.242
**Parapophysis.BV [mm³]**	0.242	±	0.151	0.153	±	0.020	0.262
**Parapophysis.BV/TV**	0.706	±	0.042	0.695	±	0.006	0.565

Supplemental Figures

S3 Table. Statistical analysis of mineralization of the precaudal and caudal vertebrae in 1-year-old zebrafish

c	CHD7 $^{+/+}$ 1-year-old			CHD7 $^{-/-}$ 1-year-old				
precaudal vertebra	n = 8			n = 8			*p(t-test)*	*p(F-test)*
**Arch.vBMD [gHA/cm^3]**	0.369	±	0.043	0.356	±	0.047	0.593	0.995
**Body.vBMD [gHA/cm^3]**	0.445	±	0.050	0.464	±	0.064	0.520	0.643
**ArchOpen.angle [°]**	20.4	±	2.5	26.2	±	10.6	0.171	0.002
**ArchRise1.angle [°]**	80.0	±	3.0	76.7	±	6.0	0.195	0.128
**ArchRise2.angle [°]**	80.4	±	2.0	77.4	±	5.7	0.198	0.019
**Diff.R1-R2 [°]**	3.9	±	2.5	4.8	±	3.9	0.618	0.361
**Body.angle [°]**	134.5	±	5.1	133.8	±	11.1	0.869	0.081
**Vertebra.length [µm]**	0.601	±	0.230	0.670	±	0.084	0.472	0.011
caudal vertebra	n = 8			n = 8			*p(t-test)*	*p(F-test)*
**Arch.vBMD [gHA/cm^3]**	0.413	±	0.020	0.433	±	0.052	0.334	0.027
**Body.vBMD [gHA/cm^3]**	0.451	±	0.022	0.486	±	0.055	0.129	0.040
**NeuralBody.angle [°]**	133.7	±	4.2	133.9	±	11.5	0.965	0.023
**NeuralArchOpen.angle [°]**	17.4	±	1.7	19.1	±	3.5	0.265	0.091
**NeuralArchRise1.angle [°]**	82.2	±	2.9	79.9	±	3.0	0.146	0.955
**NeuralArchRise2.angle [°]**	80.7	±	2.5	81.5	±	2.5	0.547	0.835
**Diff.Neural.R1-R2 [°]**	4.1	±	3.0	3.7	±	2.5	0.802	0.523
**HemalBody.angle [°]**	127.0	±	4.2	127.7	±	2.0	0.684	0.061
**HemanArchOpen.angle [°]**	20.3	±	2.2	24.2	±	4.9	0.071	0.070
**HemalArchRise1.angle [°]**	81.0	±	3.1	78.7	±	6.6	0.410	0.096
**HemalArchRise2.angle [°]**	78.3	±	2.7	77.8	±	4.1	0.788	0.378
**Diff.Hemal.R1-R2 [°]**	5.6	±	2.4	7.6	±	5.7	0.393	0.051
**Vertebra.length [µm]**	0.656	±	0.037	0.681	±	0.061	0.360	0.272

Supplemental Figures

S4 Table. Statistical analysis of mineralization of the precaudal and caudal vertebrae in 2-year-old zebrafish

	CHD7 $^{+/+}$ 2-year-old			CHD7 $^{-/-}$ 2-year-old				
precaudal vertebra	n = 5			n = 5			*p(t-test)*	*p(F-test)*
**Arch.vBMD [gHA/cm³]**	0.370	±	0.035	0.326	±	0.014	0.045	0.101
**Body.vBMD [gHA/cm³]**	0.501	±	0.036	0.523	±	0.026	0.298	0.544
**ArchOpen.angle [°]**	25.4	±	1.6	23.2	±	2.0	0.115	0.642
**ArchRise1.angle [°]**	78.4	±	2.4	79.7	±	3.6	0.558	0.440
**ArchRise2.angle [°]**	76.4	±	1.9	77.4	±	4.7	0.698	0.115
**Diff.R1-R2 [°]**	3.3	±	3.1	4.9	±	6.4	0.667	0.197
**Body.angle [°]**	123.6	±	5.7	139.8	±	4.1	0.002	0.603
**Vertebra.length [µm]**	0.704	±	0.071	0.677	±	0.044	0.503	0.460
caudal vertebra	n = 5			n = 5			*p(t-test)*	*p(F-test)*
**Arch.vBMD [gHA/cm³]**	0.498	±	0.033	0.464	±	0.020	0.096	0.333
**Body.vBMD [gHA/cm³]**	0.538	±	0.028	0.531	±	0.019	0.657	0.443
**NeuralBody.angle [°]**	124.9	±	9.5	124.8	±	2.8	0.994	0.038
**NeuralArchOpen.angle [°]**	23.6	±	2.6	22.8	±	4.5	0.741	0.308
**NeuralArchRise1.angle [°]**	77.8	±	2.8	76.0	±	2.5	0.317	0.803
**NeuralArchRise2.angle [°]**	79.0	±	2.4	81.0	±	3.0	0.268	0.656
**Diff.Neural.R1-R2 [°]**	3.1	±	2.4	5.0	±	3.4	0.327	0.517
**HemalBody.angle [°]**	120.7	±	6.5	128.7	±	2.0	0.049	0.039
**HemanArchOpen.angle [°]**	24.9	±	5.1	24.2	±	2.1	0.781	0.113
**HemalArchRise1.angle [°]**	77.6	±	3.9	76.4	±	4.4	0.657	0.825
**HemalArchRise2.angle [°]**	77.5	±	3.8	79.0	±	5.0	0.594	0.624
**Diff.Hemal.R1-R2 [°]**	4.6	±	2.3	5.5	±	7.1	0.791	0.049
**Vertebra.length [µm]**	0.721	±	0.065	0.657	±	0.039	0.105	0.335

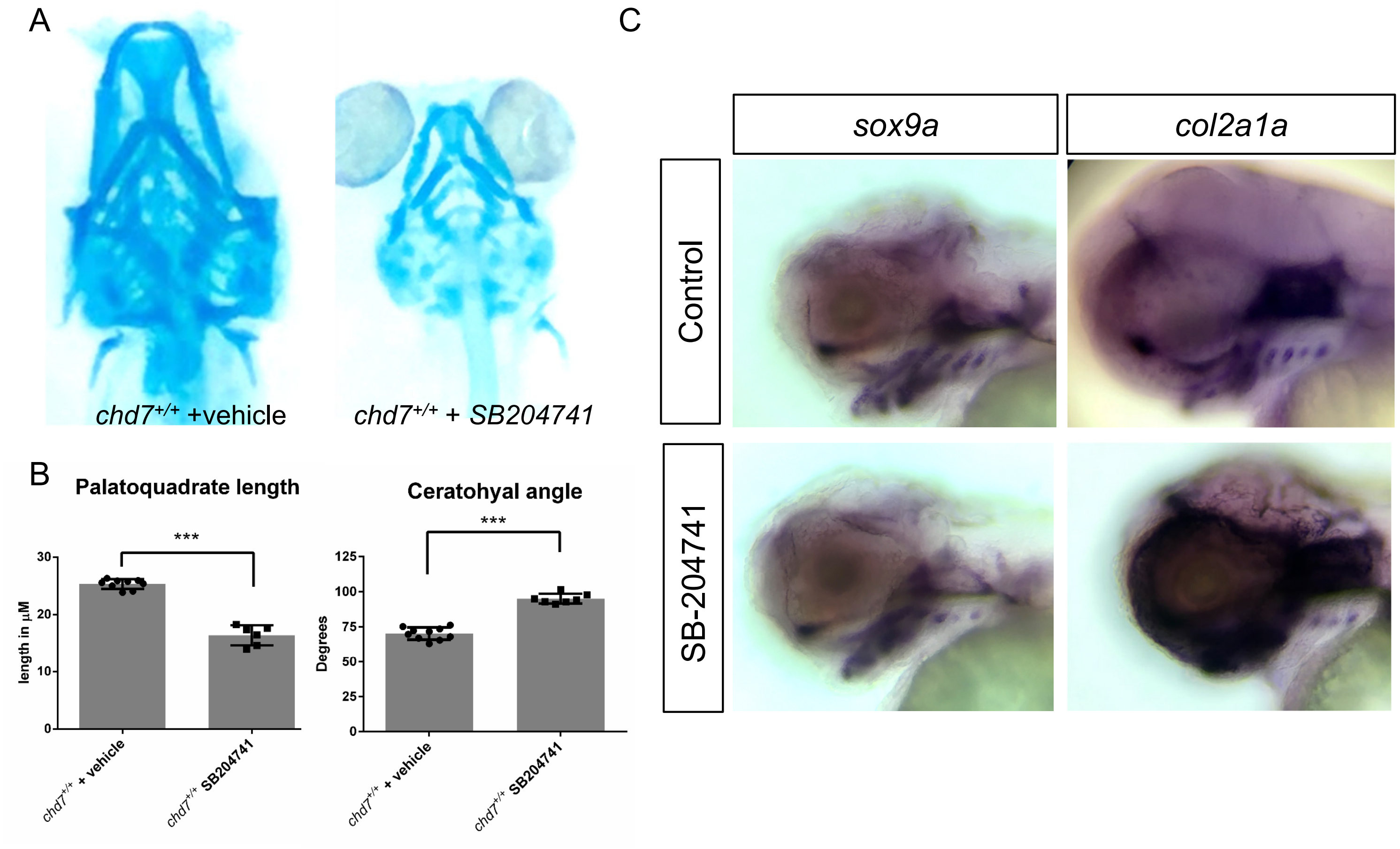

Fig S6. Craniofacial development with Htr2b inhibitor SB204741. *(A)* Alcian blue staining of 6 dpf larvae treated with vehicle or Htr2b inhibitor SB204741. *(B)* Statistical analysis of palatoquadrate length and ceratohyal angle in vehicle or inhibitor treated larvae. *(C)* WISH of *sox9a* and *col2a1a* in craniofacial regions of control or Htr2b inhibitor treated 5dpf larvae, lateral view. *** denotes $p<0.001$.

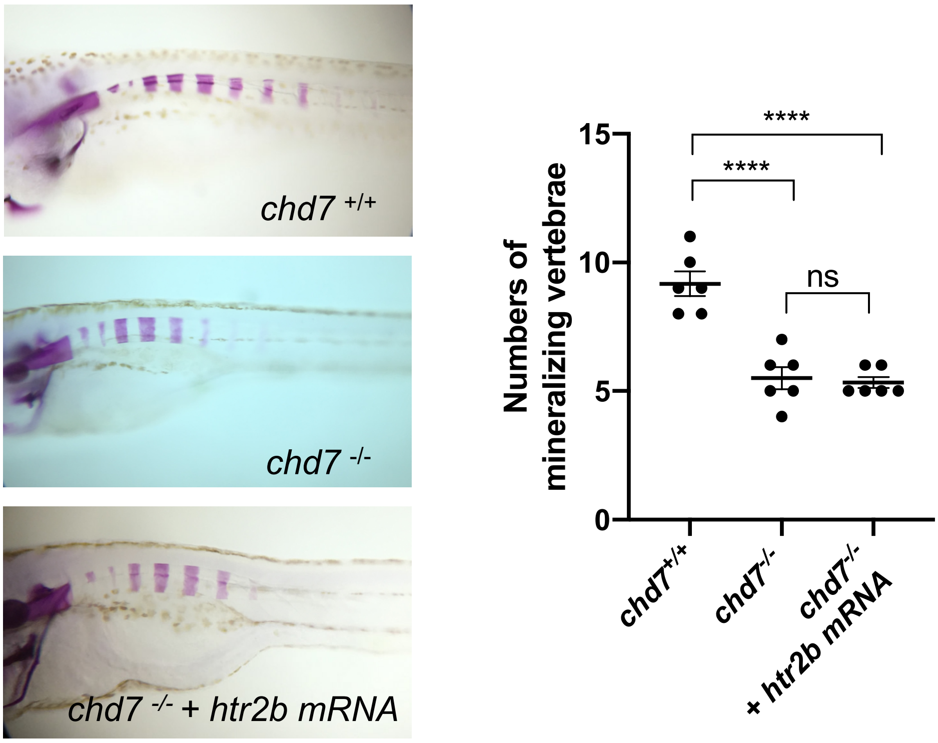

Fig S7. Rate of mineralization upon *htr2b* rescue experiments at 9dpf. *(A)* Alizarin Red staining for rate of mineralization in 9 dpf *chd7*$^{+/+}$, *chd7*$^{-/-}$ and *chd7*$^{-/-}$ + *htr2b* mRNA larvae (n=6). *(B)* Graphical representation showing no rescue of mineralization at 9dpf upon *htr2b* mRNA overexpression in *chd7*$^{-/-}$ embryos. **** denotes p<0.0001 and ns = not significant.

Supplemental Figures

S5 Table. Primer List qPCR

Target	FW Primer	RV Primer
elf1-α	GTGGCTGGAGACAGCAAGA	AGAGATCTGACCAGGGTGGTT
runx2a	TGACGTACCTGAGAGGCGT	GCAGCCGTATCCTGCATACC
runx2b	CGGCTCCTACCAGTTCTCCA	CCATCTCCCTCCACTCCTCC
mgp	ACGACACGGAGAGAAGTCCT	TGGAGGTGTTTGTGAACCCA
sost	ACGGACTTATGGAGCCTCAG	TGGAAGGCACTGACCAGAA
ctsk	TGGAACGGATCAGCAGTGTG	TCTATGCCAACTGACACGGG
acp5a	GACAGACACGCTGAGCATGAG	ACTGGTTACTTCCTTGAGCTTCCA
postna	CTGACTCAGCAAAGCAGGTG	GTTCAGTGGCGCAAGTACAG
sp7	TACCACCGGGAGGTCTTCTT	TTTACCGTACACCTTCCCGC
col2a1a	CAACACGATGTAGAGGTGGACG	CAGGGTGGCAGAGTTTCAGG
htr2b	GAGAAAACTCCAGAACGCCAC	GTGGAGTTGTAAAGCACTGTGAC
